# Supplementary material for: Host MOSPD2 enrichment at the parasitophorous vacuole membrane varies between Toxoplasma strains and involves complex interactions
Source: mSphere. 2023 Jun 21;8(4):e00670-22. doi: 10.1128/msphere.00670-22 (PMC10449529; doi:10.1128/msphere.00670-22)
Supplement: Table S1 — Primer list. [file msphere.00670-22-s0001.pdf]

Supplementary Table 1

Primer ID	Primer sequence (5' -> 3')
A1	TCCAGTGTGGTGGAATTCTGCAGATGCCACCATGGGCAAGCCCATC
A2	AGCGGCCGCCACTGTGCTGGATTTAACTGTACAATAAATAGAAGAAAGAGG
A3	CACCGACTTACAGTTCCACCCATGT
A4	AAACACATGGGTGGAACTGTAAGTC
A5	CACCGCGCGACGACTCAACCTAGTC
A6	AAACGACTAGGTTGAGTCGTCGCGC
A7	GACATAATGGCTGCAGAAATGGAACAGT
A8	AAAATCGTCTTGGGCAGAGACTGTTA
A9	GTGATTCTCTGCCATGCTAGCGGT
A10	AGCTATCCACCACTAGTAGATGAT
A11	TAACTCGAGCATGCATCTAGAGGGC
A12	GATACAACGCTGAACTTGGTCTTCA
A13	AAGTTAACGACCGAAATACCCGCGAG
A14	AAAACTCGCGGGTATTTCGGTCGTTA
A15	TTACCGTTCGTATAATGTATGCTATA
A16	ATCGAATTCTACCGTTCGTATAGCATA
A17	CATGTACTAAGTGACCGTGCATTGA
A18	TGTCTACGCTGTGCTAACATTCTATG
A19	AGCGGCCGCCACTGTGCTGGATTTAGATACAACGCTGAACTTGGT
A20	AGACACCGACTCTAGTCCAGTGTGGTGGAATTCTGCAGAT
A21	TCCAGAGGTTGATTGTCGAGCGGCCGCCACTGTGCTGGAT
A22	GCAACCAGGATTTATACAAG
A23	GACAGCACCGCTAGCATGGCAGAGAATCACGTGAGCAAGGGCGAGGAGCTGTTCA
A24	TATCTTCACTGGTTTTATCTGACATCTTGTACAGCTCGTCCATGCCGAGA
A25	TCTCGGCATGGACGAGCTGTACAAGATGTCAGATAAAACCAGTGAAGATA
A26	TTGAAAGCAGCATTCATTAACCACATCTTGTACAGCTCGTCCATGCCGA
A27	AATTGTGAAAACCTGGCTTGGTTAACTCGAGCATGCATCTAGAGGGC
A28	TGACATCTTGTACAGCTCGTCCATG
A29	ACGAGCTGTACAAGATGTCAGCTAGCATGGCAGAGAATCACGC
A30	TGATTCTCTGCCATGCTAGCTGACATCTTGTACAGCTCGTCCA
A31	GCCCTCTAGATGCATGCTCGAGTTA
A32	GCTTCCGTTCCCCCTCACGACGTTTTAGAGCTAGAAATAGC
A33	AACTTGACATCCCCATTTAC
