# Supplementary material for: Host MOSPD2 enrichment at the parasitophorous vacuole membrane varies between Toxoplasma strains and involves complex interactions
Source: mSphere. 2023 Jun 21;8(4):e00670-22. doi: 10.1128/msphere.00670-22 (PMC10449529; doi:10.1128/msphere.00670-22)
Supplement: Data Set S1 — Complete mass spectrometry data from Human and T. gondii. [file msphere.00670-22-s0002.pdf]

Gene Name	Transcript Product	Control #1	Control #2	MOSPD2 #1	MOSPD2 #2	MOSPD2 /Control
Motile sperm domain-containing pro	MOSPD2	0	0	2091	1939	2016
Vesicle-associated membrane protei	VAPA	0	0	91	81	87
Mitochondrial proton/calcium exchar	LETM1	0	0	33	39	37
Oxysterol-binding protein-related pro	OSBPL1A	0	0	25	29	28
Ras-related protein Rab-1B	RAB1B	0	0	26	25	26.5
Vesicle-associated membrane protei	VAPB	0	0	26	24	26
Signal recognition particle receptor s	SRPRA	0	0	19	19	20
Aspartyl/asparaginyl beta-hydroxylas	ASPH	0	0	26	11	19.5
Ribosome-binding protein 1	RRBP1	0	0	21	15	19
MICOS complex subunit MIC60	IMMT	0	0	16	20	19
PAT complex subunit CCDC47	CCDC47	0	0	18	16	18
Transducin beta-like protein 2	TBL2	0	0	17	17	18
Extended synaptotagmin-1	ESYT1	0	0	19	14	17.5
Sodium/potassium-transporting ATP	ATP1A1	0	0	17	16	17.5
Plasma membrane calcium-transpor	ATP2B4	0	0	18	14	17
Vesicular integral-membrane protein	LMAN2	0	0	13	18	16.5
Protein ERGIC-53	LMAN1	0	0	13	17	16
Protein wntless homolog	WLS	0	0	16	14	16
Signal transducer and activator of tra	STAT1	0	0	14	15	15.5
Lipase maturation factor 2	LMF2	0	0	11	16	14.5
Dolichyl-diphosphooligosaccharide--p	STT3A	0	0	16	10	14
Integrin alpha-2	ITGA2	0	0	15	10	13.5
Elongation factor 1-gamma	EEF1G	0	0	14	11	13.5
Syntaxin-4	STX4	0	0	12	12	13
Atlastin-3	ATL3	0	0	13	10	12.5
Transmembrane emp24 domain-con	TMED9	0	0	11	12	12.5
Membrane-associated phosphatidyli	PITPNM1	0	0	12	10	12
LEM domain-containing protein 2	LEMD2	0	0	12	9	11.5
Prohibitin	PHB	0	0	12	8	11
Catechol O-methyltransferase	COMT	0	0	10	9	10.5
Cytochrome b-c1 complex subunit 2,	UQCRC2	0	0	10	9	10.5
T-complex protein 1 subunit gamma	CCT3	0	0	9	10	10.5
Prohibitin-2	PHB2	0	0	10	9	10.5
Lysosome membrane protein 2	SCARB2	1	0	17	21	13.3333
Oxysterol-binding protein-related pro	OSBPL3	0	0	11	7	10
eIF-2-alpha kinase activator GCN1	GCN1	0	0	8	10	10
Heme oxygenase 1	HMOX1	0	0	9	9	10
Transmembrane 9 superfamily mem	TM9SF3	0	0	9	9	10
Sterol-4-alpha-carboxylate 3-dehydro	NSDHL	0	0	11	6	9.5
Adipocyte plasma membrane-associa	APMAP	0	0	11	6	9.5
CD59 glycoprotein	CD59	0	0	10	7	9.5
Torsin-1A-interacting protein 1	TOR1AIP1	0	0	10	7	9.5
Chondroitin sulfate proteoglycan 4	CSPG4	0	0	8	9	9.5

Probable glutathione peroxidase 8	GPX8	0	0	8	9	9.5
Regulator of microtubule dynamics p	RMDN3	0	0	8	9	9.5
NADPH--cytochrome P450 reductase	POR	0	0	10	6	9
Elongation factor 1-delta	EEF1D	0	0	10	6	9
Voltage-dependent anion-selective c	VDAC2	0	0	10	6	9
Secretory carrier-associated membra	SCAMP2	0	0	7	9	9
Basigin	BSG	0	0	7	9	9
Malectin	MLEC	0	0	8	8	9
Epidermal growth factor receptor	EGFR	0	0	5	10	8.5
Long-chain-fatty-acid--CoA ligase 3	ACSL3	0	0	6	9	8.5
Guanine nucleotide-binding protein G	GNAI2	0	0	6	9	8.5
Ras-related protein Ral-A	RALA	0	0	9	6	8.5
C-type mannose receptor 2	MRC2	0	0	6	9	8.5
Ras-related protein Rab-6A	RAB6A	0	0	8	7	8.5
B-cell receptor-associated protein 31	BCAP31	0	0	7	8	8.5
Basic leucine zipper and W2 domain-	BZW1	0	0	8	7	8.5
Perilipin-3	PLIN3	0	1	17	13	10.6667
Protein S100-A6	S100A6	0	0	9	5	8
Polypeptide N-acetylgalactosaminylt	GALNT2	0	0	8	6	8
Lanosterol 14-alpha demethylase	CYP51A1	0	0	8	6	8
ADP-ribosylation factor-like protein 8	ARL8B	0	0	8	6	8
Cytochrome c oxidase subunit 4 isofo	COX4I1	0	0	7	7	8
Protein LYRIC	MTDH	0	0	7	7	8
Endoplasmic reticulum-Golgi interme	ERGIC1	1	0	11	17	10
Inhibitor of nuclear factor kappa-B ki	IKBIP	0	0	4	9	7.5
ATP synthase F(0) complex subunit B	ATP5PB	0	0	5	8	7.5
Exportin-2	CSE1L	0	0	5	8	7.5
Nicalin	NCLN	0	0	8	5	7.5
Integrin alpha-V	ITGAV	0	0	6	7	7.5
Integrin alpha-5	ITGA5	0	0	7	6	7.5
Plasma membrane calcium-transport	ATP2B1	0	0	6	7	7.5
Prostaglandin G/H synthase 2	PTGS2	0	0	7	6	7.5
Ras-related protein Rab-8A	RAB8A	0	0	6	7	7.5
Prolow-density lipoprotein receptor-r	LRP1	0	0	6	7	7.5
Neutral alpha-glucosidase AB	GANAB	0	0	6	7	7.5
Translocating chain-associated mem	TRAM1	0	0	7	6	7.5
Vesicle-associated membrane protei	VAMP3	0	0	6	7	7.5
Calcium-binding mitochondrial carrie	SLC25A24	0	0	6	7	7.5
Acyl-CoA 6-desaturase	FADS2	0	0	3	9	7
Membrane-associated progesterone	PGRMC2	0	0	4	8	7
Syntaxin-7	STX7	0	0	8	4	7
Caveolin-1	CAV1	0	0	8	4	7
Thioredoxin-related transmembrane	TMX1	0	0	8	4	7
Mannosyl-oligosaccharide glucosidas	MOGS	0	0	5	7	7
Signal peptidase complex subunit 2	SPCS2	0	0	7	5	7

Serine/threonine-protein phosphatas	PGAM5	0	0	5	7	7
Ras-related protein Rab-18	RAB18	0	0	7	5	7
GTP-binding protein SAR1a	SAR1A	0	0	5	7	7
Protein PRRC2C	PRRC2C	0	0	7	5	7
Neuropilin-1	NRP1	0	0	6	6	7
Platelet-derived growth factor recept	PDGFRB	0	0	6	6	7
High affinity cationic amino acid tran	SLC7A1	0	0	6	6	7
Phosphatidylinositol-3-phosphatase S	SACM1L	0	0	6	6	7
Peptidyl-prolyl cis-trans isomerase FI	FKBP11	0	0	6	6	7
Very-long-chain enoyl-CoA reductase	TECR	0	0	6	6	7
Dehydrogenase/reductase SDR famil	DHRS7	0	0	6	6	7
Vesicle-trafficking protein SEC22b	SEC22B	3	0	25	24	10.2
Trifunctional enzyme subunit alpha, r	HADHA	1	0	12	11	8.33333
Guanine nucleotide-binding protein G	GNB1	0	0	1	10	6.5
cAMP-dependent protein kinase type	PRKAR2A	0	0	4	7	6.5
Tyrosine-protein phosphatase non-re	PTPN1	0	0	4	7	6.5
T-complex protein 1 subunit zeta	CCT6A	0	0	7	4	6.5
Matrix-remodeling-associated protei	MXRA7	0	0	4	7	6.5
Ras-related protein Rab-32	RAB32	0	0	4	7	6.5
Neutral amino acid transporter B(0)	SLC1A5	0	0	4	7	6.5
Caveolae-associated protein 3	CAVIN3	0	0	7	4	6.5
Secretory carrier-associated membra	SCAMP3	0	0	5	6	6.5
Secretory carrier-associated membra	SCAMP1	0	0	5	6	6.5
Serine/threonine-protein phosphatas	PPP2R1A	0	0	6	5	6.5
Endothelin-converting enzyme 1	ECE1	0	0	6	5	6.5
Ras-related protein Rab-1A	RAB1A	0	0	6	5	6.5
Very-long-chain 3-oxoacyl-CoA reduct	HSD17B12	0	0	6	5	6.5
Retinol dehydrogenase 11	RDH11	0	0	6	5	6.5
Translocation protein SEC63 homolog	SEC63	0	0	6	5	6.5
Voltage-dependent anion-selective c	VDAC3	0	0	6	5	6.5
NADH-cytochrome b5 reductase 3	CYB5R3	0	3	21	23	9.2
Ras-related protein Rab-3B	RAB3B	0	0	7	3	6
Guanine nucleotide-binding protein G	GNB2	0	0	7	3	6
Long-chain-fatty-acid--CoA ligase 1	ACSL1	0	0	6	4	6
Sodium/potassium-transporting ATPa	ATP1B3	0	0	6	4	6
Transmembrane 9 superfamily mem	TM9SF4	0	0	6	4	6
Transportin-1	TNPO1	0	0	4	6	6
MICOS complex subunit MIC19	CHCHD3	0	0	4	6	6
Peptidyl-tRNA hydrolase 2, mitochon	PTRH2	0	0	6	4	6
Ras-related protein Rap-1b-like prote		0	0	5	5	6
Surfeit locus protein 4	SURF4	0	0	5	5	6
STARD3 N-terminal-like protein	STARD3NL	0	0	5	5	6
Tubulin beta-4A chain	TUBB4A	0	0	5	5	6
Cation-independent mannose-6-phos	IGF2R	0	0	5	5	6
ADP-ribosylation factor GTPase-activ	ARFGAP1	0	0	5	5	6

LIM domain only protein 7	LMO7	0	0	5	5	6
Golgi apparatus protein 1	GLG1	0	0	5	5	6
Syntaxin-binding protein 3	STXBP3	0	0	6	3	5.5
Ras-related protein R-Ras2	RRAS2	0	0	3	6	5.5
Large neutral amino acids transporte	SLC7A5	0	0	3	6	5.5
Phosphatidylinositol 4-kinase type 2-a	PI4K2A	0	0	6	3	5.5
Sideroflexin-3	SFXN3	0	0	6	3	5.5
Monocarboxylate transporter 4	SLC16A3	0	0	4	5	5.5
Long-chain-fatty-acid--CoA ligase 4	ACSL4	0	0	5	4	5.5
CAAX prenyl protease 1 homolog	ZMPSTE24	0	0	4	5	5.5
Solute carrier family 2, facilitated glu	SLC2A1	0	0	5	4	5.5
Heme oxygenase 2	HMOX2	0	0	5	4	5.5
ADP-ribosylation factor-like protein 1	ARL1	0	0	5	4	5.5
Glycerol-3-phosphate dehydrogenase	GPD2	0	0	4	5	5.5
Inactive tyrosine-protein kinase 7	PTK7	0	0	4	5	5.5
Fibronectin type III domain-containin	FNDC3B	0	0	5	4	5.5
Saccharopine dehydrogenase-like oxi	SCCPDH	0	0	4	5	5.5
Dolichyl-diphosphooligosaccharide--p	STT3B	0	0	4	5	5.5
Dephospho-CoA kinase domain-conta	DCAKD	0	0	5	4	5.5
FAS-associated factor 2	FAF2	0	0	5	4	5.5
Elongation of very long chain fatty ac	ELOVL1	0	0	4	5	5.5
Sideroflexin-1	SFXN1	0	0	5	4	5.5
Ras-related protein Rab-7a	RAB7A	0	3	19	19	8
Dolichyl-diphosphooligosaccharide--p	RPN2	3	1	25	19	7.66667
60S ribosomal protein L36a	RPL36A	0	0	0	8	5
Gap junction alpha-1 protein	GJA1	0	0	2	6	5
Trifunctional enzyme subunit beta, m	HADHB	0	0	6	2	5
Proteasome activator complex subun	PSME2	0	0	2	6	5
Glyceraldehyde-3-phosphate dehydro	GAPDH	0	0	5	3	5
Protein S100-A4	S100A4	0	0	5	3	5
Alpha-1,3-mannosyl-glycoprotein 2-b	MGAT1	0	0	5	3	5
Coatomer subunit alpha	COPA	0	0	5	3	5
Ras-related protein Rab-31	RAB31	0	0	3	5	5
Protein disulfide-isomerase TMX3	TMX3	0	0	3	5	5
Endophilin-B1	SH3GLB1	0	0	3	5	5
Trans-Golgi network integral membr	TGOLN2	0	0	4	4	5
Erlin-1	ERLIN1	0	0	4	4	5
ATP synthase subunit d, mitochondria	ATP5PD	0	0	4	4	5
Myelin protein zero-like protein 1	MPZL1	0	0	4	4	5
Cytochrome c oxidase subunit 2	MT-CO2	0	0	4	4	5
Nodal modulator 3	NOMO3	0	0	4	4	5
NAD(P) transhydrogenase, mitochond	NNT	0	0	4	4	5
GPI-anchor transamidase	PIGK	0	0	4	4	5
Tubulin alpha-1C chain	TUBA1C	0	0	4	4	5
Ras-related protein Rab-21	RAB21	0	0	4	4	5

Myoferlin	MYOF	6	6	64	50	8.28571
Ras-related protein Rab-2A	RAB2A	0	2	13	12	6.75
Ras-related protein Rab-14	RAB14	2	0	12	12	6.5
ADP/ATP translocase 3	SLC25A6	3	1	22	18	7
Ras-related protein Rab-11B	RAB11B	0	2	11	12	6.25
Kallikrein-15	KLK15	0	0	0	7	4.5
HLA class I histocompatibility antiger	HLA-C	0	0	6	1	4.5
Tumor protein D54	TPD52L2	0	0	2	5	4.5
Prenylcysteine oxidase 1	PCYOX1	0	0	5	2	4.5
Protein RER1	RER1	0	0	4	3	4.5
ER lumen protein-retaining receptor	KDELR3	0	0	4	3	4.5
ATP synthase subunit g, mitochondri	ATP5MG	0	0	3	4	4.5
4F2 cell-surface antigen heavy chain	SLC3A2	0	0	3	4	4.5
Microsomal glutathione S-transferas	MGST1	0	0	3	4	4.5
Prostaglandin G/H synthase 1	PTGS1	0	0	3	4	4.5
ATP-binding cassette sub-family D m	ABCD3	0	0	4	3	4.5
Vesicle-fusing ATPase	NSF	0	0	3	4	4.5
Aldehyde dehydrogenase family 3 me	ALDH3A2	0	0	4	3	4.5
Signal peptidase complex subunit 3	SPCS3	0	0	4	3	4.5
Signal peptidase complex catalytic su	SEC11A	0	0	3	4	4.5
Antigen peptide transporter 1	TAP1	0	0	4	3	4.5
Apoptosis regulator BAX	BAX	0	0	3	4	4.5
ADP-ribosylation factor-like protein 6	ARL6IP1	0	0	4	3	4.5
ER membrane protein complex subur	EMC1	0	0	4	3	4.5
Vang-like protein 1	VANGL1	0	0	4	3	4.5
Protein FAM3C	FAM3C	0	0	4	3	4.5
Mitochondrial import receptor subun	TOMM22	0	0	3	4	4.5
40S ribosomal protein S10	RPS10	3	0	17	13	6.4
ATP synthase subunit alpha, mitocho	ATP5F1A	3	4	27	32	6.77778
Phosphate carrier protein, mitochond	SLC25A3	3	2	23	21	6.57143
Membrane-associated progesterone	PGRMC1	0	1	8	6	5.33333
Lamina-associated polypeptide 2, iso	TMPO	1	0	7	7	5.33333
Protein transport protein Sec61 subu	SEC61A1	0	2	10	11	5.75
Leucine-rich repeat-containing protei	LRRC59	3	2	22	20	6.28571
Transforming protein RhoA	RHOA	1	1	8	12	5.5
TBC1 domain family member 8	TBC1D8	0	0	6	0	4
Cytochrome c oxidase subunit 5B, mi	COX5B	0	0	5	1	4
Coiled-coil domain-containing protei	CCDC154	0	0	2	4	4
NPC intracellular cholesterol transpo	NPC1	0	0	4	2	4
Prolyl 4-hydroxylase subunit alpha-2	P4HA2	0	0	2	4	4
Cytochrome b5 type B	CYB5B	0	0	4	2	4
[F-actin]-monooxygenase MICAL2	MICAL2	0	0	4	2	4
Cytochrome b5	CYB5A	0	0	2	4	4
Cytochrome c1, heme protein, mitocl	CYC1	0	0	4	2	4
Tricarboxylate transport protein, mit	SLC25A1	0	0	4	2	4

3-beta-hydroxysteroid-Delta(8),Delta	EBP	0	0	4	2	4
Prostacyclin synthase	PTGIS	0	0	4	2	4
Protein NOXP20	FAM114A1	0	0	4	2	4
Ras-related protein Rab-34	RAB34	0	0	4	2	4
Extended synaptotagmin-2	ESYT2	0	0	3	3	4
Serine palmitoyltransferase 1	SPTLC1	0	0	3	3	4
NADH dehydrogenase [ubiquinone] ir	NDUFS3	0	0	3	3	4
Anthrax toxin receptor 2	ANTXR2	0	0	3	3	4
Protein transport protein Sec61 subu	SEC61G	0	0	3	3	4
Alpha-mannosidase 2	MAN2A1	0	0	3	3	4
Transmembrane protein 119	TMEM119	0	0	3	3	4
Ras-related protein Rab-6D	RAB6D	0	0	3	3	4
Mitochondrial antiviral-signaling prot	MAVS	0	0	3	3	4
Dedicator of cytokinesis protein 7	DOCK7	0	0	3	3	4
Transmembrane protein 165	TMEM165	0	0	3	3	4
ATPase family AAA domain-containir	ATAD3B	3	0	10	14	5.2
Aminopeptidase N	ANPEP	0	0	5	0	3.5
Tyrosine-protein phosphatase non-re	PTPN2	0	0	0	5	3.5
T-complex protein 1 subunit alpha	TCP1	0	0	0	5	3.5
CD151 antigen	CD151	0	0	0	5	3.5
T-complex protein 1 subunit delta	CCT4	0	0	0	5	3.5
Cytoplasmic dynein 1 heavy chain 1	DYNC1H1	0	0	5	0	3.5
Mitochondrial carrier homolog 2	MTCH2	0	0	5	0	3.5
Unconventional myosin-Ib	MYO1B	0	0	4	1	3.5
Heat shock 70 kDa protein 1-like	HSPA1L	0	0	4	1	3.5
Antigen peptide transporter 2	TAP2	0	0	1	4	3.5
Exostosin-2	EXT2	0	0	4	1	3.5
Endoplasmic reticulum transmembra	ATP13A1	0	0	4	1	3.5
Sodium channel protein type 10 subu	SCN10A	0	0	1	4	3.5
ATPase GET3	GET3	0	0	2	3	3.5
Transferrin receptor protein 1	TFRC	0	0	2	3	3.5
Platelet-derived growth factor recept	PDGFRA	0	0	3	2	3.5
Cytochrome c oxidase subunit 5A, mi	COX5A	0	0	3	2	3.5
Vesicle-associated membrane protei	VAMP7	0	0	2	3	3.5
CD81 antigen	CD81	0	0	3	2	3.5
Contactin-associated protein 1	CNTNAP1	0	0	2	3	3.5
Transmembrane emp24 domain-cont	TMED1	0	0	2	3	3.5
Trophoblast glycoprotein	TPBG	0	0	2	3	3.5
Metal cation symporter ZIP14	SLC39A14	0	0	3	2	3.5
Mitochondrial import inner membrar	TIMM50	0	0	3	2	3.5
LETM1 domain-containing protein 1	LETMD1	0	0	2	3	3.5
Inositol 1,4,5-trisphosphate receptor-	ITPRIP	0	0	3	2	3.5
Mitochondrial fission factor	MFF	0	0	2	3	3.5
Golgi-resident adenosine 3',5'-bisphc	BPNT2	0	0	2	3	3.5
Protein cornichon homolog 4	CNIH4	0	0	3	2	3.5

Calcium load-activated calcium chan	TMCO1	0	0	3	2	3.5
Cytochrome c oxidase assembly facto	COA3	0	0	3	2	3.5
Thioredoxin-related transmembrane	TMX2	0	0	3	2	3.5
Voltage-dependent anion-selective c	VDAC1	4	4	25	26	5.3
Integrin beta-1	ITGB1	4	4	26	24	5.2
Emerin	EMD	2	0	7	9	4.5
Calnexin	CANX	6	5	29	35	5.07692
Matrix metalloproteinase-14	MMP14	4	0	12	14	4.66667
CD44 antigen	CD44	5	4	22	28	4.72727
Heat shock protein HSP 90-beta	HSP90AB1	4	6	25	29	4.66667
Importin subunit beta-1	KPNB1	2	0	6	8	4
Mitochondrial 2-oxoglutarate/malate	SLC25A11	0	1	5	4	3.66667
Fibrocystin	PKHD1	0	0	0	4	3
Endoglin	ENG	0	0	0	4	3
Nicotinamide N-methyltransferase	NNMT	0	0	0	4	3
Signal recognition particle 54 kDa pro	SRP54	0	0	0	4	3
Transmembrane protein 214	TMEM214	0	0	4	0	3
Cullin-associated NEDD8-dissociated	CAND1	0	0	0	4	3
Protein YIPF5	YIPF5	0	0	4	0	3
Transmembrane 9 superfamily mem	TM9SF2	0	0	0	4	3
T-complex protein 1 subunit eta	CCT7	0	0	0	4	3
Transmembrane emp24 domain-con	TMED5	0	0	4	0	3
Tapasin	TAPBP	0	0	1	3	3
Squalene synthase	FDFT1	0	0	1	3	3
Methionine--tRNA ligase, cytoplasmi	MARS1	0	0	3	1	3
Syntaxin-5	STX5	0	0	1	3	3
Mas-related G-protein coupled recep	MRGPRF	0	0	1	3	3
Endoplasmic reticulum junction form	LNPK	0	0	1	3	3
Stomatin-like protein 2, mitochondri	STOML2	0	0	3	1	3
Torsin-1A	TOR1A	0	0	2	2	3
Exportin-1	XPO1	0	0	2	2	3
Signal transducer and activator of tra	STAT3	0	0	2	2	3
Signal transducer and activator of tra	STAT6	0	0	2	2	3
T-complex protein 1 subunit theta	CCT8	0	0	2	2	3
14-3-3 protein zeta/delta	YWHAZ	0	0	2	2	3
FAS-associated death domain protei	FADD	0	0	2	2	3
Peptidyl-prolyl cis-trans isomerase FI	FKBP8	0	0	2	2	3
ER membrane protein complex subu	EMC2	0	0	2	2	3
Delta(24)-sterol reductase	DHCR24	0	0	2	2	3
Synaptophysin-like protein 1	SYPL1	0	0	2	2	3
Receptor expression-enhancing prote	REEP3	0	0	2	2	3
Transmembrane protein 205	TMEM205	0	0	2	2	3
Solute carrier family 35 member F6	SLC35F6	0	0	2	2	3
ER membrane protein complex subu	MMGT1	0	0	2	2	3
Protein jagunal homolog 1	JAGN1	0	0	2	2	3

Zinc transporter SLC39A7	SLC39A7	0	0	2	2	3
Delta-sarcoglycan	SGCD	0	0	2	2	3
Lysophospholipid acyltransferase 7	MBOAT7	0	0	2	2	3
Ceroid-lipofuscinosis neuronal protei	CLN6	0	0	2	2	3
Disintegrin and metalloproteinase do	ADAM22	0	0	2	2	3
Melanoma-associated antigen D2	MAGED2	0	0	2	2	3
Tubulin beta-3 chain	TUBB3	4	2	14	18	4.25
Cytoskeleton-associated protein 4	CKAP4	9	6	36	36	4.35294
Tubulin alpha-1B chain	TUBA1B	36	0	78	88	4.42105
Signal recognition particle receptor s	SRPRB	0	11	28	25	4.23077
Sulfide:quinone oxidoreductase, mito	SQOR	3	0	5	12	3.8
HLA class I histocompatibility antigen	HLA-A	1	0	2	6	3.33333
ATP synthase subunit O, mitochondri	ATP5PO	0	1	5	3	3.33333
Transmembrane protein 33	TMEM33	1	0	3	5	3.33333
Thy-1 membrane glycoprotein	THY1	1	0	4	4	3.33333
Protein transport protein Sec61 subu	SEC61B	2	0	7	5	3.5
Sarcoplasmic/endoplasmic reticulum	ATP2A2	8	5	31	28	4.06667
Dolichyl-diphosphooligosaccharide--p	RPN1	8	13	45	48	4.13043
Transmembrane emp24 domain-con	TMED7	3	0	8	7	3.4
Hemoglobin subunit alpha	HBA2	1	0	2	5	3
Dolichol-phosphate mannosyltransfe	DPM1	0	0	0	3	2.5
Metaxin-2	MTX2	0	0	3	0	2.5
PRA1 family protein 3	ARL6IP5	0	0	3	0	2.5
NADH dehydrogenase [ubiquinone] 1	NDUFA10	0	0	0	3	2.5
Sphingosine-1-phosphate lyase 1	SGPL1	0	0	0	3	2.5
Ras-related protein R-Ras	RRAS	0	0	0	3	2.5
Metalloproteinase inhibitor 3	TIMP3	0	0	0	3	2.5
Ran GTPase-activating protein 1	RANGAP1	0	0	0	3	2.5
Actin, aortic smooth muscle	ACTA2	0	0	3	0	2.5
Voltage-dependent L-type calcium ch	CACNA1D	0	0	3	0	2.5
Proteasome activator complex subun	PSME1	0	0	3	0	2.5
Leukocyte surface antigen CD47	CD47	0	0	0	3	2.5
Squalene monooxygenase	SQLE	0	0	3	0	2.5
Serum paraoxonase/arylesterase 2	PON2	0	0	3	0	2.5
Vacuolar protein sorting-associated p	VPS13D	0	0	3	0	2.5
Coiled-coil domain-containing protein	CCDC91	0	0	3	0	2.5
Syntaxin-12	STX12	0	0	3	0	2.5
Nurim	NRM	0	0	3	0	2.5
CDGSH iron-sulfur domain-containing	CISD2	0	0	0	3	2.5
Adenosine 3'-phospho 5'-phosphosul	SLC35B2	0	0	3	0	2.5
ATP synthase membrane subunit K, r	ATP5MK	0	0	0	3	2.5
Sphingosine 1-phosphate receptor 3	S1PR3	0	0	0	3	2.5
Protein S100-A13	S100A13	0	0	3	0	2.5
Cyclin-dependent kinase 12	CDK12	0	0	3	0	2.5
SUN domain-containing protein 2	SUN2	0	0	0	3	2.5

Cell surface hyaluronidase	CEMIP2	0	0	0	3	2.5
2-hydroxyacyl-CoA lyase 2	ILVBL	0	0	2	1	2.5
Serine palmitoyltransferase 2	SPTLC2	0	0	1	2	2.5
Acyl-CoA (8-3)-desaturase	FADS1	0	0	2	1	2.5
Transforming acidic coiled-coil-conta	TACC1	0	0	2	1	2.5
Zinc finger protein-like 1	ZFPL1	0	0	1	2	2.5
Golgi SNAP receptor complex membe	GOSR1	0	0	1	2	2.5
Thioredoxin domain-containing prote	TXNDC12	0	0	2	1	2.5
Cytochrome c oxidase subunit 6C	COX6C	0	0	2	1	2.5
Myristoylated alanine-rich C-kinase s	MARCKS	0	0	2	1	2.5
Signal recognition particle 9 kDa prot	SRP9	0	0	1	2	2.5
ATP synthase subunit e, mitochondria	ATP5ME	0	0	2	1	2.5
26S proteasome regulatory subunit 8	PSMC5	0	0	1	2	2.5
Pumilio homolog 1	PUM1	0	0	1	2	2.5
Acylglycerol kinase, mitochondrial	AGK	0	0	2	1	2.5
Transmembrane protein 41B	TMEM41B	0	0	2	1	2.5
Neutral cholesterol ester hydrolase 1	NCEH1	0	0	2	1	2.5
Neuroligin-4, Y-linked	NLGN4Y	0	0	2	1	2.5
Fatty acyl-CoA reductase 1	FAR1	0	0	2	1	2.5
Ceramide synthase 2	CERS2	0	0	1	2	2.5
Chloride channel CLIC-like protein 1	CLCC1	0	0	2	1	2.5
Synaptic vesicle membrane protein V	VAT1	0	0	1	2	2.5
Selenoprotein S	SELENOS	0	0	2	1	2.5
ADP-dependent glucokinase	ADPGK	0	0	2	1	2.5
Normal mucosa of esophagus-specif	NMES1	0	0	2	1	2.5
OCIA domain-containing protein 1	OCIAD1	0	0	1	2	2.5
CKLF-like MARVEL transmembrane d	CMTM6	0	0	1	2	2.5
Glutaminyl-peptide cyclotransferase-	QPCTL	0	0	1	2	2.5
NADH-cytochrome b5 reductase 1	CYB5R1	0	0	1	2	2.5
Signal peptidase complex subunit 1	SPCS1	0	0	1	2	2.5
Tubulin beta-6 chain	TUBB6	5	5	22	20	3.66667
Transmembrane emp24 domain-cont	TMED10	7	0	16	14	3.55556
ATP synthase subunit gamma, mitoc	ATP5F1C	3	1	10	8	3.33333
Glutamine--fructose-6-phosphate am	GFPT1	4	0	8	10	3.33333
Importin-7	IPO7	1	1	6	4	3
Ras GTPase-activating-like protein IC	IQGAP1	2	2	7	10	3.16667
Ras-related protein Rab-5C	RAB5C	3	1	9	8	3.16667
Transmembrane emp24 domain-cont	TMED2	2	2	8	9	3.16667
14-3-3 protein theta	YWHAQ	2	4	11	13	3.25
Eukaryotic translation initiation facto	EIF4G2	1	0	2	4	2.66667
ATP synthase subunit beta, mitochon	ATP5F1B	4	6	17	19	3.16667
ATP-dependent 6-phosphofructokinas	PFKP	1	4	8	9	2.71429
Minor histocompatibility antigen H13	HM13	1	1	4	4	2.5
40S ribosomal protein S21	RPS21	1	0	2	3	2.33333
Disintegrin and metalloproteinase do	ADAM10	0	0	2	0	2

Etoposide-induced protein 2.4 homol	EI24	0	0	2	0	2
Sphingolipid delta(4)-desaturase DES	DEGS1	0	0	2	0	2
PRA1 family protein 2	PRAF2	0	0	2	0	2
E3 UFM1-protein ligase 1	UFL1	0	0	2	0	2
Phosphoacetylglucosamine mutase	PGM3	0	0	0	2	2
Calpain small subunit 1	CAPNS1	0	0	0	2	2
Intercellular adhesion molecule 1	ICAM1	0	0	0	2	2
Protein disulfide-isomerase	P4HB	0	0	2	0	2
Prosaposin	PSAP	0	0	0	2	2
Heat shock protein HSP 90-alpha	HSP90AA1	0	0	2	0	2
Neprilysin	MME	0	0	0	2	2
Collagen alpha-1(VI) chain	COL6A1	0	0	2	0	2
Beta-1,4-galactosyltransferase 1	B4GALT1	0	0	2	0	2
ATP synthase-coupling factor 6, mito	ATP5PF	0	0	0	2	2
Ectonucleotide pyrophosphatase/pho	ENPP1	0	0	0	2	2
Guanine nucleotide-binding protein s	GNA11	0	0	0	2	2
Translocator protein	TSPO	0	0	2	0	2
Cytochrome b-c1 complex subunit 1,	UQCRC1	0	0	0	2	2
Sterol O-acyltransferase 1	SOAT1	0	0	2	0	2
60S ribosomal protein L4	RPL4	0	0	0	2	2
Eukaryotic translation initiation facto	EIF1AX	0	0	0	2	2
NADH dehydrogenase [ubiquinone] fl	NDUFV1	0	0	0	2	2
Ras-related protein Rab-9A	RAB9A	0	0	0	2	2
Importin subunit alpha-1	KPNA2	0	0	2	0	2
ATP synthase subunit f, mitochondria	ATP5MF	0	0	2	0	2
Ras-related protein Rap-1A	RAP1A	0	0	2	0	2
Dermcidin	DCD	0	0	2	0	2
Rho-related GTP-binding protein Rho	RHOG	0	0	0	2	2
Schlafen family member 5	SLFN5	0	0	2	0	2
26S proteasome non-ATPase regulat	PSMD2	0	0	0	2	2
Disintegrin and metalloproteinase do	ADAM9	0	0	2	0	2
Polycystin-2	PKD2	0	0	0	2	2
NADH dehydrogenase [ubiquinone] 1	NDUFA9	0	0	2	0	2
Protein YIF1B	YIF1B	0	0	0	2	2
Acyl-CoA-binding domain-containing	ACBD5	0	0	2	0	2
Ankyrin repeat domain-containing pr	ANKRD13A	0	0	2	0	2
Transmembrane protein 230	TMEM230	0	0	2	0	2
Cleft lip and palate transmembrane	CLPTM1L	0	0	0	2	2
Endoplasmic reticulum-Golgi interme	ERGIC2	0	0	2	0	2
Myeloid-associated differentiation m	MYADM	0	0	2	0	2
Mitogen-activated protein kinase kin	MAP3K5	0	0	2	0	2
Transmembrane protein 70, mitocho	TMEM70	0	0	2	0	2
Derlin-1	DERL1	0	0	0	2	2
Magnesium transporter protein 1	MAGT1	0	0	0	2	2
Plasminogen receptor (KT)	PLGRKT	0	0	2	0	2

ER membrane protein complex subur	EMC7	0	0	0	2	2
Serine incorporator 1	SERINC1	0	0	0	2	2
Bcl-2-associated transcription factor	BCLAF1	0	0	2	0	2
Dolichol-phosphate mannosyltransfe	DPM3	0	0	0	2	2
Guanine nucleotide-binding protein (	GNG12	0	0	0	2	2
Vesicle transport through interaction	VTI1B	0	0	0	2	2
N-acetyl-D-glucosamine kinase	NAGK	0	0	0	2	2
Syntaxin-8	STX8	0	0	0	2	2
RuvB-like 2	RUVBL2	0	0	2	0	2
RuvB-like 1	RUVBL1	0	0	0	2	2
Chromatin target of PRMT1 protein	CHTOP	0	0	2	0	2
Sorting and assembly machinery com	SAMM50	0	0	0	2	2
UbiA prenyltransferase domain-conta	UBIAD1	0	0	2	0	2
Dolichyl-phosphate beta-glucosyltran	ALG5	0	0	0	2	2
Testis-expressed protein 264	TEX264	0	0	0	2	2
NADH dehydrogenase [ubiquinone] ir	NDUFS8	0	0	1	1	2
Importin-5	IPO5	0	0	1	1	2
Cytochrome b-c1 complex subunit 8	UQCRQ	0	0	1	1	2
Small glutamine-rich tetratricopeptic	SGTA	0	0	1	1	2
39S ribosomal protein L33, mitochon	MRPL33	0	0	1	1	2
Galactosylgalactosylxylosylprotein 3-	B3GAT3	0	0	1	1	2
Immunoglobulin heavy variable 3-43I	IGHV3-43D	0	0	1	1	2
Ras-related protein Ral-B	RALB	0	0	1	1	2
Cytochrome c oxidase subunit 7A2, m	COX7A2	0	0	1	1	2
ER lumen protein-retaining receptor	KDELR1	0	0	1	1	2
ATP synthase subunit delta, mitochor	ATP5F1D	0	0	1	1	2
Translocon-associated protein subuni	SSR1	0	0	1	1	2
Cysteine--tRNA ligase, cytoplasmic	CARS1	0	0	1	1	2
Coatomer subunit beta	COPB1	0	0	1	1	2
Beta-2-microglobulin	B2M	0	0	1	1	2
ORM1-like protein 2	ORMDL2	0	0	1	1	2
Plasma membrane ascorbate-depend	CYBRD1	0	0	1	1	2
ORM1-like protein 3	ORMDL3	0	0	1	1	2
Divergent protein kinase domain 2A	DIPK2A	0	0	1	1	2
Solute carrier family 2, facilitated glu	SLC2A14	0	0	1	1	2
Leucine-rich repeat-containing protei	LRRC15	0	0	1	1	2
Protein FAM162A	FAM162A	0	0	1	1	2
Mitochondrial import receptor subun	TOMM6	0	0	1	1	2
Charged multivesicular body protein (	CHMP6	0	0	1	1	2
DDRGK domain-containing protein 1	DDRGK1	0	0	1	1	2
Sodium-coupled neutral amino acid t	SLC38A2	0	0	1	1	2
EF-hand calcium-binding domain-cor	CRACR2A	0	0	1	1	2
Transmembrane protein 109	TMEM109	0	0	1	1	2
Anthrax toxin receptor 1	ANTXR1	0	0	1	1	2
Cell cycle control protein 50A	TMEM30A	0	0	1	1	2

NADH dehydrogenase [ubiquinone] 1	NDUFA13	0	0	1	1	2
Testis-expressed protein 2	TEX2	3	6	14	13	2.63636
Caveolae-associated protein 1	CAVIN1	4	0	7	6	2.5
Reticulon-4	RTN4	5	3	14	10	2.6
CTP synthase 1	CTPS1	2	0	5	2	2.25
Nicotinamide phosphoribosyltransfer	NAMPT	3	1	7	5	2.33333
Ras-related protein Rab-10	RAB10	4	2	9	8	2.375
Clathrin heavy chain 1	CLTC	6	0	10	7	2.375
D-3-phosphoglycerate dehydrogenase	PHGDH	5	4	14	10	2.36364
CD99 antigen	CD99	0	1	1	3	2
Dihydropyrimidinase-related protein	DPYSL3	1	0	1	3	2
Cytochrome c oxidase subunit NDUFA	NDUFA4	1	0	2	2	2
Dolichyl-diphosphooligosaccharide--p	DDOST	6	3	12	11	2.27273
Actin, alpha cardiac muscle 1	ACTC1	39	14	71	57	2.36364
40S ribosomal protein S19	RPS19	8	4	13	17	2.28571
Ras-related C3 botulinum toxin subst	RAC1	8	4	17	12	2.21429
Heat shock protein beta-1	HSPB1	2	2	5	5	2
Tubulin beta-4B chain	TUBB4B	4	5	10	9	1.90909
Protein S100-A10	S100A10	5	2	3	12	1.88889
Dolichyl-diphosphooligosaccharide--p	DAD1	2	3	6	5	1.85714
Heat shock 70 kDa protein 1B	HSPA1B	3	1	5	4	1.83333
Cathepsin B	CTSB	0	1	0	3	1.66667
Proteasomal ubiquitin receptor ADRM	ADRM1	1	0	2	1	1.66667
ADP-ribosylation factor-like protein 6	ARL6IP4	0	1	1	2	1.66667
Immunoglobulin heavy variable 3-64	IGHV3-64	0	0	1	0	1.5
Transmembrane protein 223	TMEM223	0	0	0	1	1.5
Alkyldihydroxyacetonephosphate synt	AGPS	0	0	0	1	1.5
Stearoyl-CoA desaturase	SCD	0	0	0	1	1.5
Prostaglandin E synthase	PTGES	0	0	0	1	1.5
Microsomal glutathione S-transferas	MGST3	0	0	0	1	1.5
Centromere/kinetochore protein zw1	ZW10	0	0	0	1	1.5
Beta-1,4-galactosyltransferase 4	B4GALT4	0	0	1	0	1.5
Mannose-P-dolichol utilization defect	MPDU1	0	0	0	1	1.5
Tubby-related protein 3	TULP3	0	0	0	1	1.5
Vesicle-associated membrane protei	VAMP5	0	0	1	0	1.5
Reticulon-3	RTN3	0	0	0	1	1.5
NADH dehydrogenase [ubiquinone] 1	NDUFC2	0	0	1	0	1.5
Acyl-protein thioesterase 2	LYPLA2	0	0	0	1	1.5
NADH dehydrogenase [ubiquinone] 1	NDUFB10	0	0	1	0	1.5
Mitochondrial import receptor subun	TOMM40	0	0	0	1	1.5
Putative HLA class I histocompatibilit	HLA-H	0	0	0	1	1.5
Apolipoprotein C-III	APOC3	0	0	1	0	1.5
Apolipoprotein D	APOD	0	0	1	0	1.5
Integrin beta-3	ITGB3	0	0	1	0	1.5
Neurofilament medium polypeptide	NEFM	0	0	0	1	1.5

Rho-related GTP-binding protein Rho	RHOC	0	0	0	1	1.5
Golgi pH regulator B	GPR89B	0	0	0	1	1.5
60 kDa SS-A/Ro ribonucleoprotein	RO60	0	0	1	0	1.5
Macrophage migration inhibitory fact	MIF	0	0	1	0	1.5
Glucosidase 2 subunit beta	PRKCSH	0	0	1	0	1.5
Endoplasmin	HSP90B1	0	0	0	1	1.5
Cytochrome c oxidase subunit 6B1	COX6B1	0	0	0	1	1.5
26S proteasome regulatory subunit 6	PSMC3	0	0	0	1	1.5
Cation-dependent mannose-6-phospl	M6PR	0	0	0	1	1.5
Succinate dehydrogenase [ubiquinone	SDHB	0	0	0	1	1.5
14-3-3 protein beta/alpha	YWHAB	0	0	1	0	1.5
Profilin-2	PFN2	0	0	0	1	1.5
Catenin alpha-1	CTNNA1	0	0	1	0	1.5
Catenin beta-1	CTNNB1	0	0	1	0	1.5
26S proteasome regulatory subunit 7	PSMC2	0	0	0	1	1.5
Matrin-3	MATR3	0	0	0	1	1.5
Lanosterol synthase	LSS	0	0	1	0	1.5
Adhesion G protein-coupled receptor	ADGRE5	0	0	1	0	1.5
Puromycin-sensitive aminopeptidase	NPEPPS	0	0	0	1	1.5
Integrin alpha-1	ITGA1	0	0	0	1	1.5
Synaptojanin-2-binding protein	SYNJ2BP	0	0	0	1	1.5
Cell division control protein 42 homo	CDC42	0	0	1	0	1.5
Ras-related protein Rab-5B	RAB5B	0	0	0	1	1.5
Guanine nucleotide-binding protein G	GNG5	0	0	0	1	1.5
Interferon-induced transmembrane p	IFITM3	0	0	1	0	1.5
Induced myeloid leukemia cell differe	MCL1	0	0	0	1	1.5
Sequestosome-1	SQSTM1	0	0	0	1	1.5
Major facilitator superfamily domain	MFSD10	0	0	0	1	1.5
Delta(14)-sterol reductase LBR	LBR	0	0	0	1	1.5
Methylsterol monooxygenase 1	MSMO1	0	0	1	0	1.5
Beta-sarcoglycan	SGCB	0	0	0	1	1.5
Nucleolar MIF4G domain-containing	NOM1	0	0	1	0	1.5
Rab-like protein 3	RABL3	0	0	0	1	1.5
ER membrane protein complex subur	EMC4	0	0	0	1	1.5
Schlafen family member 13	SLFN13	0	0	0	1	1.5
Dehydrogenase/reductase SDR famil	DHRS7B	0	0	1	0	1.5
Calcium-binding mitochondrial carrie	SLC25A25	0	0	1	0	1.5
Long-chain fatty acid transport prote	SLC27A4	0	0	1	0	1.5
Cytochrome P450 20A1	CYP20A1	0	0	1	0	1.5
Glycerol-3-phosphate acyltransferase	GPAT4	0	0	1	0	1.5
Ras-specific guanine nucleotide-relea	RALGPS2	0	0	1	0	1.5
Histone-arginine methyltransferase C	CARM1	0	0	0	1	1.5
ELMO domain-containing protein 2	ELMOD2	0	0	0	1	1.5
Outer mitochondrial transmembrane	ATAD1	0	0	0	1	1.5
Calcium uniporter protein, mitochond	MCU	0	0	0	1	1.5

Golgin subfamily A member 5	GOLGA5	0	0	0	1	1.5
tRNA-splicing endonuclease subunit S	TSEN15	0	0	0	1	1.5
U4/U6 small nuclear ribonucleoprote	PRPF31	0	0	1	0	1.5
Lysophosphatidic acid receptor 1	LPAR1	0	0	0	1	1.5
Nectin-2	NECTIN2	0	0	1	0	1.5
Ninjurin-1	NINJ1	0	0	0	1	1.5
Protein RFT1 homolog	RFT1	0	0	0	1	1.5
Vacuole membrane protein 1	VMP1	0	0	1	0	1.5
Solute carrier family 35 member E1	SLC35E1	0	0	0	1	1.5
26S proteasome non-ATPase regulato	PSMD1	0	0	0	1	1.5
HIG1 domain family member 2A, mit	HIGD2A	0	0	1	0	1.5
Uridine-cytidine kinase 2	UCK2	0	0	0	1	1.5
SRA stem-loop-interacting RNA-bind	SLIRP	0	0	1	0	1.5
Methyltransferase-like protein 9	METTL9	0	0	0	1	1.5
Thioredoxin-related transmembrane	TMX4	0	0	0	1	1.5
Protein Wnt-5b	WNT5B	0	0	0	1	1.5
BolA-like protein 2	BOLA2B	0	0	0	1	1.5
Phosphatidylinositol glycan anchor bi	PIGU	0	0	1	0	1.5
Regulator of nonsense transcripts 2	UPF2	0	0	0	1	1.5
Prolactin regulatory element-binding	PREB	0	0	1	0	1.5
Protein MANBAL	MANBAL	0	0	1	0	1.5
Oligosaccharyltransferase complex s	OSTC	0	0	1	0	1.5
Lysosomal cobalamin transport escor	LMBRD1	0	0	0	1	1.5
Mitochondrial carrier homolog 1	MTCH1	0	0	1	0	1.5
NADH dehydrogenase [ubiquinone] 1	NDUFAF4	0	0	0	1	1.5
Protein sel-1 homolog 1	SEL1L	0	0	0	1	1.5
ATP-binding cassette sub-family F m	ABCF2	0	0	1	0	1.5
Translocon-associated protein subun	SSR3	0	0	0	1	1.5
Integral membrane protein 2B	ITM2B	0	0	1	0	1.5
Choline-phosphate cytidylyltransfera	PCYT1B	0	0	1	0	1.5
Selenoprotein K	SELENOK	0	0	0	1	1.5
Probable ATP-dependent RNA helicas	DDX49	0	0	1	0	1.5
Tubulin beta chain	TUBB	60	57	111	123	1.98319
60S ribosomal protein L17	RPL17	10	0	14	5	1.75
TAR DNA-binding protein 43	TARDBP	4	0	6	2	1.66667
40S ribosomal protein S16	RPS16	29	29	54	50	1.76667
Paired mesoderm homeobox protein	PRRX1	3	0	4	2	1.6
60S ribosomal protein L22-like 1	RPL22L1	3	0	3	3	1.6
Protein-L-isoaspartate(D-aspartate) O	PCMT1	3	3	3	8	1.625
Crk-like protein	CRKL	2	4	7	4	1.625
Histone H2B type 1-N	H2BC15	6	0	7	4	1.625
40S ribosomal protein S15	RPS15	5	4	7	9	1.63636
UDP-glucose 6-dehydrogenase	UGDH	4	1	3	6	1.57143
Lysosome-associated membrane glyc	LAMP1	3	2	5	4	1.57143
Translocon-associated protein subun	SSR4	4	3	4	8	1.55556

DNA-dependent protein kinase cataly	PRKDC	14	14	23	23	1.6
40S ribosomal protein S14	RPS14	15	7	18	18	1.58333
Galectin-1	LGALS1	11	3	10	13	1.5625
28S ribosomal protein S12, mitochon	MRPS12	4	2	5	5	1.5
Protein disulfide-isomerase A3	PDIA3	6	6	10	9	1.5
40S ribosomal protein S5	RPS5	13	14	18	24	1.51724
Tubulin beta-2A chain	TUBB2A	4	5	9	5	1.45455
Poly(rC)-binding protein 1	PCBP1	3	4	7	4	1.44444
Transgelin-2	TAGLN2	2	1	2	3	1.4
Nucleosome-remodeling factor subu	BPTF	3	0	0	5	1.4
Guanine nucleotide-binding protein-l	GNL3	0	1	2	0	1.33333
60S ribosomal protein L29	RPL29	1	0	1	1	1.33333
Transcription elongation factor A pro	TCEAL5	1	0	1	1	1.33333
60S ribosomal protein L27a	RPL27A	8	4	10	8	1.42857
Endoplasmic reticulum chaperone Bi	HSPA5	22	14	28	25	1.44737
F-actin-capping protein subunit alpha	CAPZA1	10	3	11	8	1.4
Calponin-3	CNN3	2	2	4	2	1.33333
Heterogeneous nuclear ribonucleopro	HNRNPM	13	13	17	19	1.35714
Pleckstrin homology domain-containi	PLEKHA2	5	3	7	4	1.3
Peroxisomal 2,4-dienoyl-CoA reducta	DECR2	3	5	6	5	1.3
Elongation factor Tu, mitochondrial	TUFM	1	1	3	0	1.25
S-phase kinase-associated protein 1	SKP1	1	1	2	1	1.25
GRB10-interacting GYF protein 1	GIGYF1	0	2	3	0	1.25
Fatty acid synthase	FASN	14	14	16	21	1.3
60 kDa heat shock protein, mitochond	HSPD1	10	15	14	19	1.2963
Cysteine and glycine-rich protein 2	CSRP2	12	10	17	12	1.29167
60S ribosomal protein L22	RPL22	5	8	9	8	1.26667
Protein pelota homolog	PELO	4	2	5	3	1.25
Parkinson disease protein 7	PARK7	6	8	9	9	1.25
Zinc finger CCCH domain-containing p	ZC3H7B	7	0	4	5	1.22222
Peroxiredoxin-2	PRDX2	3	0	2	2	1.2
SEC23-interacting protein	SEC23IP	91	80	108	102	1.22543
Heat shock cognate 71 kDa protein	HSPA8	34	41	45	47	1.22078
Dihydropyrimidinase-related protein	DPYSL2	3	5	3	7	1.2
Immunoglobulin heavy variable 3/OR	IGHV3OR16-9	6	2	6	4	1.2
Peroxiredoxin-4	PRDX4	47	35	51	48	1.20238
Peroxiredoxin-1	PRDX1	19	20	26	21	1.19512
Serine/arginine-rich splicing factor 3	SRSF3	1	3	3	2	1.16667
40S ribosomal protein S29	RPS29	10	10	11	13	1.18182
Polypyrimidine tract-binding protein	PTBP1	6	4	5	7	1.16667
40S ribosomal protein S17	RPS17	21	14	17	24	1.16216
Ketosamine-3-kinase	FN3KRP	8	3	7	6	1.15385
ADP-ribosylation factor 4	ARF4	3	2	4	2	1.14286
Dynein light chain 1, cytoplasmic	DYNLL1	2	3	3	3	1.14286
Annexin A2	ANXA2	32	30	36	36	1.15625

Ankyrin repeat and KH domain-contai	ANKHD1	11	8	8	14	1.14286
DNA-3-methyladenine glycosylase	MPG	16	15	18	17	1.12121
Phenylalanine--tRNA ligase alpha sul	FARSA	5	2	5	3	1.11111
Alpha-aminoadipic semialdehyde del	ALDH7A1	4	3	4	4	1.11111
40S ribosomal protein S4, Y isoform 2	RPS4Y2	3	4	4	4	1.11111
Guanine nucleotide-binding protein s	GNA12	49	0	29	26	1.11765
40S ribosomal protein S26	RPS26	13	11	13	14	1.11538
Prelamin-A/C	LMNA	5	3	6	3	1.1
40S ribosomal protein S15a	RPS15A	8	10	8	12	1.1
Putative sodium-coupled neutral ami	SLC38A10	28	22	32	23	1.09615
60S ribosomal protein L9	RPL9P9	5	7	5	8	1.07143
40S ribosomal protein S23	RPS23	20	21	24	20	1.06977
Ubiquitin-60S ribosomal protein L40	UBA52	8	5	3	11	1.06667
Eukaryotic translation initiation facto	EIF4E2	10	5	8	8	1.05882
Elongation factor 1-alpha 1	EEF1A1	75	73	73	81	1.04
Protein disulfide-isomerase A6	PDIA6	17	13	15	16	1.03125
40S ribosomal protein S18	RPS18	32	37	40	31	1.02817
60S ribosomal protein L38	RPL38	54	54	62	47	1.00909
Poly(rC)-binding protein 2	PCBP2	1	1	0	2	1
Ribonucleoprotein PTB-binding 1	RAVER1	1	1	0	2	1
Heterogeneous nuclear ribonucleopro	HNRNPA3	3	1	0	4	1
Eukaryotic initiation factor 4A-I	EIF4A1	2	3	5	0	1
40S ribosomal protein S13	RPS13	24	12	21	15	1
40S ribosomal protein S7	RPS7	12	10	12	10	1
Annexin A1	ANXA1	8	7	11	4	1
DNA replication licensing factor MCM	MCM3	8	6	10	4	1
E3 ubiquitin-protein ligase SHPRH	SHPRH	8	8	8	8	1
Plasminogen activator inhibitor 1 RN	SERBP1	5	4	4	5	1
Microtubule-associated protein 1B	MAP1B	5	3	4	4	1
Myeloid differentiation primary respo	MYD88	5	2	4	3	1
40S ribosomal protein S12	RPS12	3	4	4	3	1
60S ribosomal protein L13	RPL13	3	4	4	3	1
40S ribosomal protein S6	RPS6	4	2	2	4	1
S-adenosylmethionine synthase isofo	MAT2A	4	1	2	3	1
Laminin subunit beta-3	LAMB3	3	0	3	0	1
Matrix-remodeling-associated protei	MXRA5	0	3	3	0	1
Signal recognition particle 14 kDa pro	SRP14	2	2	2	2	1
Non-histone chromosomal protein HI	HMGN2	2	0	2	0	1
RNA transcription, translation and tra	RTRAF	2	0	2	0	1
Sperm-associated antigen 5	SPAG5	1	1	1	1	1
60S ribosomal protein L34	RPL34	1	0	0	1	1
Protein LSM12 homolog	LSM12	1	0	1	0	1
DnaJ homolog subfamily C member 1	DNAJC10	0	1	0	1	1
RNA-binding protein 42	RBM42	0	1	1	0	1
Histone H1.2	H1-2	14	0	6	8	1

Arginine and glutamate-rich protein	ARGLU1	5	0	4	1	1
Ubiquitin-40S ribosomal protein S27a	RPS27A	3	0	1	2	1
Active regulator of SIRT1	RPS19BP1	3	0	2	1	1
Microtubule-actin cross-linking facto	MACF1	92	96	92	94	0.98947
40S ribosomal protein S3a	RPS3A	38	34	36	35	0.98649
60S ribosomal protein L11	RPL11	30	31	33	27	0.98413
Protein Smaug homolog 1	SAMD4A	66	44	51	57	0.98214
40S ribosomal protein S2	RPS2	61	44	48	55	0.98131
GRB10-interacting GYF protein 2	GIGYF2	19	16	18	16	0.97297
Valine--tRNA ligase	VARS1	132	106	114	117	0.97083
Thioredoxin	TXN	8	9	7	9	0.94737
60S ribosomal protein L23a	RPL23A	17	16	15	16	0.94286
ADP/ATP translocase 2	SLC25A5	9	5	6	7	0.9375
Peptidyl-prolyl cis-trans isomerase A	PPIA	8	6	7	6	0.9375
Protein transport protein Sec23A	SEC23A	7	5	6	5	0.92857
Receptor of activated protein C kinas	RACK1	5	6	3	7	0.92308
Histone H2A type 1-J	H2AC14	11	0	6	4	0.92308
Serpin H1	SERPINH1	24	21	22	19	0.91489
Putative 60S ribosomal protein L39-li	RPL39P5	0	10	0	9	0.91667
Eukaryotic translation initiation facto	EIF4G3	6	4	5	4	0.91667
40S ribosomal protein S3	RPS3	83	76	70	74	0.90683
40S ribosomal protein S20	RPS20	36	26	31	25	0.90625
Plectin	PLEC	130	108	102	113	0.90417
60S ribosomal protein L3	RPL3	5	4	6	2	0.90909
ATPase family AAA domain-containir	ATAD3A	6	3	3	5	0.90909
Mitogen-activated protein kinase 1	MAPK1	6	2	4	3	0.9
40S ribosomal protein SA	RPSA	4	4	5	2	0.9
Sodium/bile acid cotransporter 5	SLC10A5	11	6	7	8	0.89474
40S ribosomal protein S4, X isoform	RPS4X	56	51	48	47	0.88991
Pyruvate kinase PKM	PKM	3	4	3	3	0.88889
Palladin	PALLD	7	15	12	7	0.875
Thioredoxin domain-containing prote	TXNDC5	29	23	22	23	0.87037
Heterogeneous nuclear ribonucleopro	HNRNPL	9	5	8	4	0.875
Tensin-3	TNS3	21	15	13	18	0.86842
Constitutive coactivator of PPAR-gam	FAM120A	15	6	9	9	0.86957
Transcription factor jun-D	JUND	2	4	2	3	0.875
F-actin-capping protein subunit beta	CAPZB	8	5	5	6	0.86667
Transforming growth factor-beta-ind	TGFBI	7	6	7	4	0.86667
ATP-dependent RNA helicase DDX3X	DDX3X	70	55	54	53	0.85827
40S ribosomal protein S11	RPS11	69	62	56	56	0.85714
Elongin-A	ELOA	17	16	17	11	0.85714
Caldesmon	CALD1	22	18	17	16	0.83333
Vimentin	VIM	79	78	79	51	0.83019
Splicing factor, proline- and glutamir	SFPQ	17	11	12	11	0.83333
Destrin	DSTN	10	6	6	7	0.83333

Probable ATP-dependent RNA helicas	DDX17	14	13	13	9	0.82759
Voltage-dependent L-type calcium ch	CACNB2	74	93	79	57	0.81657
Elongin-C	ELOC	4	0	3	0	0.83333
Polymerase delta-interacting protein	POLDIP3	2	2	2	1	0.83333
PDZ and LIM domain protein 4	PDLIM4	4	0	1	2	0.83333
Cofilin-1	CFL1	8	6	4	7	0.8125
40S ribosomal protein S27	RPS27	11	8	7	8	0.80952
Actin, cytoplasmic 1	ACTB	9	35	18	17	0.80435
Protein PRRC2B	PRRC2B	138	118	99	102	0.78682
Histone H1.5	H1-5	8	9	6	7	0.78947
60S ribosomal protein L23	RPL23	39	36	30	28	0.77922
DNA topoisomerase 1	TOP1	22	17	15	15	0.78049
60S ribosomal protein L21	RPL21	13	8	10	6	0.78261
40S ribosomal protein S24	RPS24	28	15	16	17	0.77778
Signal-induced proliferation-associat	SIPA1L3	46	42	31	36	0.76667
Ig-like domain-containing protein (Fr		599	594	461	445	0.75983
Probable ATP-dependent RNA helicas	DDX5	69	69	48	56	0.75714
UBX domain-containing protein 1	UBXN1	2	1	2	0	0.8
ATP-dependent RNA helicase DDX18	DDX18	3	0	2	0	0.8
Probable ATP-dependent RNA helicas	DDX6	2	1	1	1	0.8
DNA replication licensing factor MCM	MCM5	11	0	3	5	0.76923
Uncharacterized protein C15orf39	C15orf39	4	6	3	4	0.75
Trinucleotide repeat-containing gene	TNRC6C	4	6	3	4	0.75
60S ribosomal protein L30	RPL30	11	10	7	8	0.73913
Plasminogen activator inhibitor 2	SERPINB2	16	8	8	9	0.73077
Transitional endoplasmic reticulum A	VCP	20	18	13	14	0.725
Microtubule-associated protein 4	MAP4	5	1	3	1	0.75
Calponin-2	CNN2	2	4	2	2	0.75
Methyltransferase-like 26	METTL26	9	4	5	4	0.73333
Heterogeneous nuclear ribonucleopro	HNRNPH1	25	18	15	15	0.71111
Heterogeneous nuclear ribonucleopro	HNRNPU	45	35	26	30	0.70732
RNA-splicing ligase RtcB homolog	RTCB	9	3	3	5	0.71429
Transmembrane glycoprotein NMB	GPNMB	8	4	5	3	0.71429
PAN2-PAN3 deadenylation complex s	PAN3	9	6	4	6	0.70588
40S ribosomal protein S9	RPS9	19	11	10	10	0.6875
60S ribosomal protein L37a	RPL37A	21	12	10	12	0.68571
Myelin-associated neurite-outgrowth	FAM168B	1	1	1	0	0.75
Tropomyosin alpha-3 chain	TPM3	1	1	1	0	0.75
Polymerase delta-interacting protein	POLDIP2	1	1	0	1	0.75
40S ribosomal protein S4, Y isoform	RPS4Y1	2	0	1	0	0.75
RNA-binding motif, single-stranded-i	RBMS1	2	0	0	1	0.75
KH domain-containing, RNA-binding,	KHDRBS1	2	0	1	0	0.75
Translation machinery-associated pro	TMA16	2	0	1	0	0.75
GTP-binding nuclear protein Ran	RAN	2	3	1	2	0.71429
Phosphatidylinositol-binding clathrin	PICALM	3	2	1	2	0.71429

Muscleblind-like protein 3	MBNL3	3	2	2	1	0.71429
40S ribosomal protein S28	RPS28	2	6	2	3	0.7
ELAV-like protein 1	ELAVL1	6	2	3	2	0.7
High mobility group nucleosome-bind	HMGN4	5	3	2	3	0.7
Serpin B8	SERPINB8	4	4	3	2	0.7
60S ribosomal protein L24	RPL24	8	3	5	2	0.69231
Immunoglobulin heavy variable 3-64I	IGHV3-64D	241	219	151	145	0.64502
RNA-binding protein 39	RBM39	6	4	3	3	0.66667
CREB-regulated transcription coactiv	CRTC3	2	5	2	2	0.66667
Nucleophosmin	NPM1	4	3	2	2	0.66667
Myosin light polypeptide 6	MYL6	4	8	4	3	0.64286
60S ribosomal protein L10	RPL10	14	11	8	7	0.62963
Liprin-alpha-1	PPFIA1	6	11	2	8	0.63158
SH3 and PX domain-containing prote	SH3PXD2B	9	8	6	4	0.63158
Heterogeneous nuclear ribonucleopro	HNRNPK	3	1	0	2	0.66667
Calmodulin-1	CALM1	2	2	1	1	0.66667
Uncharacterized protein C11orf98	C11orf98	4	0	1	1	0.66667
F-actin-capping protein subunit alpha	CAPZA2	2	7	3	2	0.63636
Protein FRG1	FRG1	6	5	3	3	0.61538
40S ribosomal protein S25	RPS25	74	68	39	42	0.57639
Mitotic checkpoint protein BUB3	BUB3	6	0	3	0	0.625
CDKN2A-interacting protein	CDKN2AIP	6	7	5	2	0.6
Neuroblast differentiation-associated	AHNAK	169	146	98	78	0.56151
Peptidyl-prolyl cis-trans isomerase B	PPIB	19	23	15	8	0.56818
Histone H4	H4-16	10	9	4	6	0.57143
Wiskott-Aldrich syndrome protein fa	WASF3	0	10	5	0	0.58333
BUB3-interacting and GLEBS motif-c	ZNF207	1	0	0	0	0.66667
Catenin delta-1	CTNND1	1	0	0	0	0.66667
H/ACA ribonucleoprotein complex su	DKC1	1	0	0	0	0.66667
Zinc finger BED domain-containing p	ZBED4	0	1	0	0	0.66667
Slit homolog 2 protein	SLIT2	1	0	0	0	0.66667
CD63 antigen	CD63	1	0	0	0	0.66667
Tenascin	TNC	1	0	0	0	0.66667
Transcriptional enhancer factor TEF-1	TEAD1	1	0	0	0	0.66667
Nuclear pore complex protein Nup15	NUP153	0	1	0	0	0.66667
Calponin-1	CNN1	1	0	0	0	0.66667
Serine/threonine-protein phosphatas	PPP2R2A	0	1	0	0	0.66667
60S ribosomal protein L6	RPL6	1	0	0	0	0.66667
Tyrosine-protein phosphatase non-re	PTPN12	0	1	0	0	0.66667
Cell division cycle protein 20 homolog	CDC20	0	1	0	0	0.66667
E3 ubiquitin-protein ligase TRIM32	TRIM32	1	0	0	0	0.66667
Serine/threonine-protein kinase PRP4	PRPF4B	0	1	0	0	0.66667
SNW domain-containing protein 1	SNW1	0	1	0	0	0.66667
Peroxisome assembly factor 2	PEX6	1	0	0	0	0.66667
Prostaglandin E synthase 3	PTGES3	0	1	0	0	0.66667

Coiled-coil domain-containing protei	CCDC183	0	1	0	0	0.66667
Hydroxysteroid dehydrogenase-like p	HSDL2	1	0	0	0	0.66667
	APOBEC3F	0	1	0	0	0.66667
Leucine-rich repeat-containing protei	LRRC47	0	1	0	0	0.66667
Protein LSM14 homolog A	LSM14A	1	0	0	0	0.66667
Cell migration-inducing and hyaluron	CEMIP	0	1	0	0	0.66667
U4/U6.U5 small nuclear ribonucleop	SNRNP27	1	0	0	0	0.66667
Heterogeneous nuclear ribonucleopro	HNRNPLL	1	0	0	0	0.66667
Coiled-coil domain-containing protei	CCDC124	0	1	0	0	0.66667
Kinesin-like protein KIF20B	KIF20B	1	0	0	0	0.66667
Programmed cell death protein 2-like	PDCD2L	0	1	0	0	0.66667
EMILIN-2	EMILIN2	1	0	0	0	0.66667
2-(3-amino-3-carboxypropyl)histidine	DPH1	1	0	0	0	0.66667
182 kDa tankyrase-1-binding protein	TNKS1BP1	1	0	0	0	0.66667
Breast carcinoma-amplified sequenc	BCAS3	1	0	0	0	0.66667
Fanconi anemia group E protein	FANCE	1	0	0	0	0.66667
Putative 40S ribosomal protein S10-l	RPS10P5	0	1	0	0	0.66667
PDZ and LIM domain protein 7	PDLIM7	0	1	0	0	0.66667
Muscleblind-like protein 1	MBNL1	0	1	0	0	0.66667
	APOBEC3C	1	0	0	0	0.66667
LanC-like protein 2	LANCL2	1	0	0	0	0.66667
SPATS2-like protein	SPATS2L	1	0	0	0	0.66667
H/ACA ribonucleoprotein complex su	NHP2	1	0	0	0	0.66667
DNA helicase MCM8	MCM8	1	0	0	0	0.66667
Ribosomal RNA-processing protein 7	RRP7A	0	1	0	0	0.66667
Protein FAM32A	FAM32A	0	1	0	0	0.66667
Nuclear receptor corepressor 2	NCOR2	0	1	0	0	0.66667
Elongin-B	ELOB	1	2	0	1	0.6
Stromal membrane-associated prote	SMAP1	2	1	0	1	0.6
Biglycan	BGN	2	3	2	0	0.57143
Phostensin	PPP1R18	2	3	2	0	0.57143
Inactive ubiquitin carboxyl-terminal h	USP53	3	2	2	0	0.57143
Ataxin-2	ATXN2	2	5	1	2	0.55556
Fibroblast growth factor 2	FGF2	4	3	2	1	0.55556
A-kinase anchor protein 2	AKAP2	5	4	4	0	0.54545
Protein RCC2	RCC2	6	3	2	2	0.54545
tRNA-splicing endonuclease subunit S	TSEN34	4	5	2	2	0.54545
Polyadenylate-binding protein 1	PABPC1	7	6	2	4	0.53333
Filamin-A	FLNA	17	13	8	6	0.5
Myosin-9	MYH9	50	52	40	8	0.48077
ATP-dependent RNA helicase DDX3Y	DDX3Y	6	6	2	3	0.5
5'-3' exoribonuclease 2	XRN2	12	11	4	6	0.48
Non-POU domain-containing octame	NONO	17	13	7	6	0.46875
Tensin-1	TNS1	31	45	20	13	0.44872
Protein PRRC2A	PRRC2A	11	13	7	3	0.46154

60S ribosomal protein L28	RPL28	3	3	1	1	0.5
Putative uncharacterized protein LOC		2	2	0	1	0.5
Lipoma-preferred partner	LPP	2	2	0	1	0.5
14-3-3 protein gamma	YWHAG	4	0	0	1	0.5
60S ribosomal protein L31	RPL31	11	13	5	4	0.42308
Phenylalanine--tRNA ligase beta sub	FARSB	6	1	0	2	0.44444
Transcription factor jun-B	JUNB	2	5	0	2	0.44444
60S ribosomal protein L35	RPL35	9	6	1	4	0.41176
60S ribosomal protein L36a-like	RPL36AL	7	9	5	0	0.38889
60S ribosomal protein L39	RPL39	28	0	9	0	0.36667
Mapk-regulated corepressor-interact	MCRIP1	1	1	0	0	0.5
DNA-directed RNA polymerase, mito	POLRMT	1	1	0	0	0.5
High mobility group protein HMGI-C	HMGA2	1	1	0	0	0.5
Ubiquitin-associated protein 2	UBAP2	1	1	0	0	0.5
Neuropeptide S receptor	NPSR1	1	1	0	0	0.5
Protein S100-A16	S100A16	1	1	0	0	0.5
Telomerase RNA component interact	TRIR	1	1	0	0	0.5
Probable ATP-dependent RNA helicas	DDX56	1	1	0	0	0.5
Zinc finger protein 593	ZNF593	2	0	0	0	0.5
Protein transport protein Sec24B	SEC24B	0	2	0	0	0.5
Protein S100-A8	S100A8	2	0	0	0	0.5
Ribonuclease inhibitor	RNH1	2	0	0	0	0.5
Casein kinase I isoform epsilon	CSNK1E	2	0	0	0	0.5
60S ribosomal protein L18	RPL18	0	2	0	0	0.5
Protein RRP5 homolog	PDCD11	2	0	0	0	0.5
Rab-like protein 6	RABL6	2	0	0	0	0.5
Putative transferase CAF17, mitocho	IBA57	2	0	0	0	0.5
Transcription initiation factor TFIID s	TAF2	2	0	0	0	0.5
Ataxin-2-like protein	ATXN2L	2	0	0	0	0.5
Ribosomal RNA processing protein 3	RRP36	2	0	0	0	0.5
Zinc finger CCCH-type antiviral protei	ZC3HAV1L	2	0	0	0	0.5
PH and SEC7 domain-containing prot	PSD3	0	2	0	0	0.5
Protein inturned	INTU	2	0	0	0	0.5
40S ribosomal protein S27a	RPS27AP5	0	2	0	0	0.5
Ubiquitin carboxyl-terminal hydrolase	USP45	4	1	1	0	0.42857
Kinetochore protein NDC80 homolog	NDC80	3	2	1	0	0.42857
Stress-70 protein, mitochondrial	HSPA9	6	5	3	0	0.38462
Elongation factor 2	EEF2	11	5	0	4	0.33333
Myosin regulatory light chain 12A	MYL12A	4	2	1	0	0.375
Phosphoenolpyruvate carboxykinase	PCK2	2	4	1	0	0.375
Leydig cell tumor 10 kDa protein hom	C19orf53	2	4	1	0	0.375
Glucose-6-phosphate 1-dehydrogena	G6PD	3	3	1	0	0.375
Centrosomal AT-AC splicing factor	CENATAC	3	3	1	0	0.375
Histone H2B type 1-O	H2BC17	1	9	2	0	0.33333
Fibronectin	FN1	39	20	12	3	0.27869

Eukaryotic translation initiation facto	EIF1AY	1	2	0	0	0.4
Histone H3.3C	H3-5	1	2	0	0	0.4
Probable non-functional immunoglob	IGHV3-35	3	0	0	0	0.4
Unconventional myosin-Id	MYO1D	3	0	0	0	0.4
Histone H2A.Z	H2AZ1	3	0	0	0	0.4
Regulator of chromosome condensat	RCC1	3	0	0	0	0.4
Peroxiredoxin-6	PRDX6	3	0	0	0	0.4
Cyclin-dependent kinase 17	CDK17	3	0	0	0	0.4
Filamin-C	FLNC	3	0	0	0	0.4
Fibrous sheath-interacting protein 2	FSIP2	3	0	0	0	0.4
RRP12-like protein	RRP12	3	0	0	0	0.4
Extracellular serine/threonine protei	FAM20C	0	3	0	0	0.4
Far upstream element-binding prote	KHSRP	0	3	0	0	0.4
Phosphatidylinositol 4-phosphate 5-k	PIP5K1A	0	3	0	0	0.4
Anillin	ANLN	0	3	0	0	0.4
Coronin-1C	CORO1C	0	3	0	0	0.4
Trinucleotide repeat-containing gene	TNRC6B	0	3	0	0	0.4
Histone H1.0	H1-0	6	5	1	1	0.30769
Double-stranded RNA-specific adeno	ADAR	11	9	4	0	0.27273
Zinc finger protein 106	ZNF106	8	5	2	0	0.26667
Src substrate cortactin	CTTN	1	3	0	0	0.33333
Actin, cytoplasmic 2	ACTG1	2	2	0	0	0.33333
Single-stranded DNA-binding protein	SSBP2	2	2	0	0	0.33333
Histone deacetylase 5	HDAC5	2	2	0	0	0.33333
Paired amphipathic helix protein Sin3	SIN3B	0	4	0	0	0.33333
High mobility group protein HMG-I/H	HMGA1	4	0	0	0	0.33333
rRNA 2'-O-methyltransferase fibrilla	FBL	4	0	0	0	0.33333
Megakaryocyte-associated tyrosine-p	MATK	4	0	0	0	0.33333
Transcription factor A, mitochondrial	TFAM	4	0	0	0	0.33333
ATP-dependent RNA helicase DDX1	DDX1	4	0	0	0	0.33333
YTH domain-containing family protei	YTHDF1	4	0	0	0	0.33333
Nucleolar GTP-binding protein 1	GTPBP4	4	0	0	0	0.33333
Tudor-interacting repair regulator pro	NUDT16L1	10	9	0	3	0.2381
Aurora kinase A	AURKA	4	1	0	0	0.28571
Putative RNA-binding protein Luc7-lik	LUC7L2	4	1	0	0	0.28571
Protein SREK1IP1	SREK1IP1	3	2	0	0	0.28571
Cystatin-B	CSTB	5	0	0	0	0.28571
SH3KBP1-binding protein 1	SHKBP1	0	5	0	0	0.28571
Protein kinase C and casein kinase su	PACSIN2	0	5	0	0	0.28571
Protein SON	SON	4	2	0	0	0.25
Nucleolar protein 6	NOL6	2	4	0	0	0.25
Axonemal dynein light intermediate	DNALI1	3	3	0	0	0.25
Heterogeneous nuclear ribonucleopro	HNRNPH2	3	3	0	0	0.25
40S ribosomal protein S8	RPS8	3	3	0	0	0.25
Guanine nucleotide-binding protein s	GNB4	3	3	0	0	0.25

Nitric oxide synthase-interacting prot	NOSIP	3	3	0	0	0.25
Histone H2A type 2-C	H2AC20	0	6	0	0	0.25
Histone H1.10	H1-10	8	5	1	0	0.2
Soluble scavenger receptor cysteine-	SSC5D	12	10	2	0	0.16667
Collagen alpha-2(VI) chain	COL6A2	5	2	0	0	0.22222
Protein FAM98A	FAM98A	2	5	0	0	0.22222
Tissue factor pathway inhibitor 2	TFPI2	4	3	0	0	0.22222
Vasorin	VASN	4	3	0	0	0.22222
60S ribosomal protein L26-like 1	RPL26L1	7	1	0	0	0.2
60S ribosomal protein L18a	RPL18A	4	4	0	0	0.2
EH domain-containing protein 2	EHD2	4	4	0	0	0.2
60S ribosomal protein L32	RPL32	6	3	0	0	0.18182
Titin	TTN	9	0	0	0	0.18182
Collagen alpha-3(VI) chain	COL6A3	13	6	1	0	0.14286
RNA cytidine acetyltransferase	NAT10	5	5	0	0	0.16667
Vacuolar protein sorting-associated p	VPS13C	10	0	0	0	0.16667
D-aspartate oxidase	DDO	8	5	0	0	0.13333
Laminin subunit beta-4	LAMB4	13	0	0	0	0.13333
Histone H1.3	H1-3	3	12	0	0	0.11765
Constitutive coactivator of PPAR-gam	FAM120C	11	7	0	0	0.1
Immunoglobulin heavy variable 3-15	IGHV3-15	19	0	0	0	0.09524
F-BAR and double SH3 domains prote	FCHSD1	0	31	0	0	0.06061
Tubulin alpha-1A chain	TUBA1A	0	35	0	0	0.05405
Vacuolar protein sorting-associated p	VPS13B	35	0	0	0	0.05405
Endothelial PAS domain-containing p	EPAS1	0	45	0	0	0.04255
E3 ubiquitin-protein ligase RNF6	RNF6	0	52	0	0	0.03704
Coiled-coil domain-containing protei	CCDC87	52	46	0	0	0.02
Voltage-dependent L-type calcium ch	CACNB3	47	51	0	0	0.02

Rank	*T. gondii* Gene ID	Transcript Product	Control #1	Control #2	MOSPD2 #1	MOSPD2 #2	MOSPD2 /Control
1	TGME49_258580	ROP17	0	0	34	31	33.5
2	TGME49_309590	ROP1	0	1	32	27	20.3333
3	TGME49_243800	long-chain fatty acid Co	0	0	12	14	14
4	TGME49_288650	GRA12	0	0	12	12	13
5	TGME49_308090	ROP5	1	0	18	15	11.6667
6	TGME49_230980	myosin I	0	0	9	9	10
7	TGME49_205250	ROP18	0	0	10	7	9.5
8	TGME49_310000	membrane protein, pu	0	0	6	8	8
9	TGME49_247350	thioredoxin domain-co	0	0	7	6	7.5
10	TGME49_233480	SRS29C	0	0	7	5	7
11	TGME49_288380	heat shock protein HSP	0	0	7	5	7
12	TGME49_311720	chaperonin protein BiP	0	0	6	5	6.5
13	TGME49_279100	MAF1a	0	1	10	6	6
14	TGME49_239740	GRA14	0	0	5	4	5.5
15	TGME49_205658	F5/8 type C domain-co	0	0	8	0	5
16	TGME49_211040	Sec61beta family prote	0	0	5	3	5
17	TGME49_236890	GRA37	0	0	2	6	5
18	TGME49_271050	SAG-related sequence	2	0	8	9	4.75
19	TGME49_210820	a ROP	0	0	4	3	4.5
20	TGME49_263700	ribosomal protein RPS1	0	0	3	4	4.5
21	TGME49_227620	GRA2	4	2	18	14	4.25
22	TGME49_288500	FAD Malate-dehydroge	0	0	0	6	4
23	TGME49_249900	adenine nucleotide tra	0	0	3	3	4
24	TGME49_269980	preprotein translocase	0	0	3	3	4
25	TGME49_295110	ROP7	0	0	2	4	4
26	TGME49_309560	nmda receptor glutam	0	0	2	4	4
27	TGME49_289690	glyceraldehyde-3-phos	0	1	6	4	4
28	TGME49_257060	translation initiation fa	0	0	5	0	3.5
29	TGME49_201390	in parasite ER?	0	0	3	2	3.5
30	TGME49_275470	GRA15	0	0	3	2	3.5
31	TGME49_306030	glutathione s-transfera	0	0	2	3	3.5
32	TGME49_308020	SAG-related sequence	0	0	3	2	3.5
33	TGME49_310750	emp24/gp25L/p24 fam	0	0	3	2	3.5
34	TGME49_319340	GRA52 (CST5)	0	0	2	3	3.5
35	TGME49_203310	GRA7	3	3	13	10	3.125
36	TGME49_288830	NADH dehydrogenase (	0	0	4	0	3
37	TGME49_214320	facilitative glucose tra	0	0	2	2	3
38	TGME49_215220	GRA22	0	0	3	1	3
39	TGME49_220240	GRA31	0	0	3	1	3
40	TGME49_297880	GRA23	0	0	3	1	3
41	TGME49_315320	SAG-related sequence	0	0	3	1	3
42	TGME49_227280	GRA3	2	2	8	8	3
43	TGME49_233460	SRS29B	10	11	30	28	2.6087
44	TGME49_204530	MIC11	0	0	3	0	2.5
45	TGME49_219320	acid phosphatase GAP	0	0	0	3	2.5
46	TGME49_248460	ubiquitin, putative	0	0	3	0	2.5
47	TGME49_259040	hypothetical protein	0	0	3	0	2.5
48	TGME49_285870	SRS20A	0	0	0	3	2.5
49	TGME49_295125	ROP4	0	0	2	1	2.5
50	TGME49_310790	CST9	0	0	1	2	2.5

51	TGME49_323100	hypothetical protein	0	0	2	1	2.5
52	TGME49_263050	ribosomal protein RPL1	2	0	5	3	2.5
53	TGME49_234450	ribosomal protein RPS1	5	6	17	10	2.23077
54	TGME49_262960	U1 snRNP-associated p	3	3	8	7	2.125
55	TGME49_214575	hypothetical protein	0	0	2	0	2
56	TGME49_251540	GRA9	0	0	0	2	2
57	TGME49_269190	glyceraldehyde-3-phos	0	0	0	2	2
58	TGME49_278660	P-type ATPase4, putati	0	0	2	0	2
59	TGME49_318190	phosphoglycerate muta	0	0	2	0	2
60	TGME49_200360	YFP -> DG	0	0	1	1	2
61	TGME49_207170	hypothetical protein	0	0	1	1	2
62	TGME49_240810	hypothetical protein	0	0	1	1	2
63	TGME49_247440	GRA33	0	0	1	1	2
64	TGME49_263300	eukaryotic porin protei	0	0	1	1	2
65	TGME49_258130	Rab1 protein	1	0	2	2	2
66	TGME49_309120	ribosomal protein RPL4	0	2	3	3	2
67	TGME49_263040	ribosomal protein RPS1	6	5	13	11	2
68	TGME49_248480	ribosomal protein RPS9	6	6	14	10	1.85714
69	TGME49_215775	ROP8	4	5	13	5	1.81818
70	TGME49_209030	actin ACT1	5	7	10	10	1.57143
71	TGME49_250810	ribosomal protein RPL3	5	4	6	9	1.54545
72	TGME49_203720	vitamin k epoxide redu	0	0	1	0	1.5
73	TGME49_221480	a MIC	0	0	1	0	1.5
74	TGME49_221620	beta-tubulin, putative	0	0	1	0	1.5
75	TGME49_225120	hypothetical protein	0	0	1	0	1.5
76	TGME49_235470	myosin A	0	0	1	0	1.5
77	TGME49_238380	hypothetical protein	0	0	0	1	1.5
78	TGME49_242070	cAMP-dependent prote	0	0	1	0	1.5
79	TGME49_248340	GTP-binding nuclear pr	0	0	1	0	1.5
80	TGME49_254720	GRA8	0	0	1	0	1.5
81	TGME49_257530	transporter, major faci	0	0	1	0	1.5
82	TGME49_258660	ROP6	0	0	0	1	1.5
83	TGME49_271888	3-ketoacyl-CoA reducta	0	0	1	0	1.5
84	TGME49_290700	GRA25	0	0	1	0	1.5
85	TGME49_310420	hypothetical protein	0	0	0	1	1.5
86	TGME49_313020	STAS domain-containir	0	0	1	0	1.5
87	TGME49_315770	cytochrome p450 supe	0	0	0	1	1.5
88	TGME49_316400	alpha tubulin TUBA1	0	0	1	0	1.5
89	TGME49_212290	ribosomal protein RPS1	6	9	11	12	1.47059
90	TGME49_289750	ribosomal-ubiquitin pr	2	6	9	3	1.4
91	TGME49_305520	ribosomal protein RPS2	8	7	8	13	1.35294
92	TGME49_243690	hypothetical protein	0	1	2	0	1.33333
93	TGME49_273760	heat shock protein HSP	4	2	5	3	1.25
94	TGME49_294800	elongation factor 1-alp	4	3	4	5	1.22222
95	TGME49_270380	ribosomal protein RPS1	8	5	7	9	1.2
96	TGME49_300190	ribosomal protein RPL3	3	2	3	3	1.14286
97	TGME49_232300	ribosomal protein RPS3	6	8	9	7	1.125
98	TGME49_249270	protein disulfide isome	3	4	3	5	1.11111
99	TGME49_213350	ribosomal protein RPS1	9	9	9	11	1.1
100	TGME49_232710	ribosomal protein RPS3	18	15	19	17	1.08571
101	TGME49_266070	ribosomal protein RPL3	5	6	6	6	1.07692
102	TGME49_262670	ribosomal protein RPL1	8	7	9	7	1.05882
103	TGME49_318440	helicase associated do	1	1	2	0	1

104	TGME49_243570	ribosomal protein RPS2	4	3	3	4	1
105	TGME49_245460	ribosomal protein RPS8	4	3	4	3	1
106	TGME49_246160	hypothetical protein	0	1	1	0	1
107	TGME49_315570	hypothetical protein	3	2	3	2	1
108	TGME49_225080	ribosomal protein RPS1	22	16	22	15	0.975
109	TGME49_223050	ribosomal protein RPS2	7	9	7	8	0.94444
110	TGME49_215460	ribosomal protein RPS2	7	3	4	5	0.91667
111	TGME49_207840	ribosomal protein RPS1	8	6	6	6	0.875
112	TGME49_261240	histone H3	5	1	5	0	0.875
113	TGME49_229250	ribosomal protein RPL2	6	0	0	5	0.875
114	TGME49_284560	ribosomal protein RPL9	3	3	4	1	0.875
115	TGME49_229670	ribosomal protein RPS2	10	12	10	8	0.83333
116	TGME49_204020	ribosomal protein RPL8	6	4	3	5	0.83333
117	TGME49_291590	hypothetical protein	4	0	0	3	0.83333
118	TGME49_299050	ribosomal protein RPL1	6	2	3	3	0.8
119	TGME49_239760	ribosomal protein RPL2	6	5	4	4	0.76923
120	TGME49_231080	ribosomal protein RPL3	16	12	11	10	0.76667
121	TGME49_248390	ribosomal protein RPL2	12	6	6	7	0.75
122	TGME49_215980	CD8 T cell antigen	1	1	1	0	0.75
123	TGME49_231140	ribosomal protein RPS2	17	8	6	11	0.7037
124	TGME49_313990	CBF/Mak21 family pro	196	191	150	117	0.69152
125	TGME49_242330	ribosomal protein RPS5	14	13	10	8	0.68966
126	TGME49_207440	ribosomal protein RPS4	45	31	21	29	0.66667
127	TGME49_268200	RNA recognition motif	7	3	5	1	0.66667
128	TGME49_230180	GRA24	3	4	3	1	0.66667
129	TGME49_289970	hypothetical protein	3	1	0	2	0.66667
130	TGME49_310490	ribosomal protein RPL2	3	1	1	1	0.66667
131	TGME49_207460	Rab5B protein	0	1	0	0	0.66667
132	TGME49_226240	bud site selection prote	1	0	0	0	0.66667
133	TGME49_234190	serine hydroxymethyltr	0	1	0	0	0.66667
134	TGME49_310740	hypothetical protein	1	0	0	0	0.66667
135	TGME49_320670	vacuolar protein sortin	1	0	0	0	0.66667
136	TGME49_315610	hypothetical protein	9	3	3	4	0.64286
137	TGME49_243460	hypothetical protein	2	4	0	3	0.625
138	TGME49_267400	ribosomal protein RPL3	31	20	18	13	0.62264
139	TGME49_226970	ribosomal protein RPS1	11	12	6	7	0.6
140	TGME49_236650	DEAD (Asp-Glu-Ala-As	16	9	6	7	0.55556
141	TGME49_244320	ribosomal protein RPL2	3	4	3	0	0.55556
142	TGME49_226250	DEAD (Asp-Glu-Ala-As	26	29	15	14	0.54386
143	TGME49_288720	ribosomal protein RPL1	4	6	4	0	0.5
144	TGME49_265090	hypothetical protein	2	2	0	1	0.5
145	TGME49_209910	histone H2Bv	0	2	0	0	0.5
146	TGME49_213890	Myb family DNA-bindir	2	0	0	0	0.5
147	TGME49_226830	DnaK family protein	2	0	0	0	0.5
148	TGME49_261250	histone H2A1	2	0	0	0	0.5
149	TGME49_278530	multiprotein bridging f	2	0	0	0	0.5
150	TGME49_282070	hypothetical protein	2	0	0	0	0.5
151	TGME49_310430	Hsp90 domain-contain	2	0	0	0	0.5
152	TGME49_312175	hypothetical protein	2	0	0	0	0.5
153	TGME49_239100	ribosomal protein RPS1	8	5	2	3	0.46667
154	TGME49_245680	ribosomal protein RPL2	6	5	1	3	0.46154
155	TGME49_291850	ribosomal protein RPS3	6	8	5	0	0.4375
156	TGME49_238010	ribosomal protein RPL2	4	1	0	1	0.42857

157	TGME49_260380	hypothetical protein	2	1	0	0	0.4
158	TGME49_203630	ribosomal protein RPL4	3	0	0	0	0.4
159	TGME49_291090	SWI2/SNF2-containing	0	3	0	0	0.4
160	TGME49_294290	Der1ER1	3	0	0	0	0.4
161	TGME49_262050	rhoptry kinase family p	5	5	0	2	0.33333
162	TGME49_210690	ribosomal protein RPS6	4	0	0	0	0.33333
163	TGME49_220490	pre-rRNA-processing p	29	32	17	0	0.30159
164	TGME49_244880	DNA-directed RNA poly	0	5	0	0	0.28571
165	TGME49_278110	1,3-beta-glucan syntha	5	0	0	0	0.28571
166	TGME49_246990	hypothetical protein	0	7	0	0	0.22222
167	TGME49_254620	ribosomal protein RPL3	10	8	0	0	0.1
168	TGME49_203520	hypothetical protein	12	7	0	0	0.09524
