# Supplementary material for: Host MOSPD2 enrichment at the parasitophorous vacuole membrane varies between Toxoplasma strains and involves complex interactions
Source: mSphere. 2023 Jun 21;8(4):e00670-22. doi: 10.1128/msphere.00670-22 (PMC10449529; doi:10.1128/msphere.00670-22)
Supplement: Supplemental Legends — Legends for Data Set S1 and Table S1. [file msphere.00670-22-s0003.pdf]

Supplementary Table 1: Primers

Supplementary Data Set 1: V5-MOSPD2 IP Ranked protein list

Complete mass spectrometry data from Human and *T. gondii*.
